# Malaria vaccines: high-throughput tools for antigens discovery with potential for their development

**Published:** 2013-06-30

**Authors:** Nora Céspedes, Andrés Vallejo, Myriam Arévalo-Herrera, Sócrates Herrera

**Affiliations:** a International Vaccine Center. E-mail: ncespedes@inmuno.org; b Universidad del Valle. E-mail: marevalo@inmuno.org

**Keywords:** malaria, vaccines, *Plasmodium*, Antigens, peptides, recombinant proteins

## Abstract

Malaria is a disease induced by parasites of the *Plasmodium* genus, which are transmitted by *Anopheles* mosquitoes and represents a great socio-economic burden Worldwide. *Plasmodium* vivax is the second species of malaria Worldwide, but it is the most prevalent in Latin America and other regions of the planet. It is currently considered that vaccines represent a cost-effective strategy for controlling transmissible diseases and could complement other malaria control measures; however, the chemical and immunological complexity of the parasite has hindered development of effective vaccines. Recent availability of several genomes of *Plasmodium* species, as well as bioinformatic tools are allowing the selection of large numbers of proteins and analysis of their immune potential. Herein, we review recently developed strategies for discovery of novel antigens with potential for malaria vaccine development.

## Abstract (es)

La malaria es una de las enfermedades transmisibles de mayor impacto socio-económico a escala mundial, y es inducida por parásitos del género *Plasmodium* transmitidos por mosquitos del genero *Anopheles*. El *Plasmodium* *vivax *ocupa el segundo lugar en prevalencia mundial, pero es la especie más frecuente en América Latina y otras regiones del planeta. Se considera que las vacunas representan una estrategia costo-efectiva para el control de enfermedades transmisibles y que podrían complementar las demás medidas de control de la malaria; sin embargo, la complejidad química e inmunológica del parasito han dificultado el desarrollo de vacunas efectivas. La reciente accesibilidad a los genomas de varias especies de *Plasmodium*, y el desarrollo de herramientas bioinformáticas están permitiendo la selección de numerosas proteínas y el análisis de su potencial inmunológico. Aquí revisamos las estrategias recientes para el descubrimiento de nuevos antígenos para el desarrollo vacunas contra la malaria.

## Epidemiological importance of malaria

Malaria represents a global public health problem that hinders socio-economic development in vast regions of the world, particularly of the planet´s Tropical and sub-Tropical areas. It is calculated that ~3-billion people from over 100 countries are exposed to the infection by one or more *Plasmodium* ^1^ species and it is estimated that in 2010 there were over 216-million clinical cases, over 650.000 of which were lethal. *P. falciparum *is the most abundant and virulent species, followed by *P. vivax *, which although producing lower mortality causes incapacitating and recurrent disease^2^. *Plasmodium. vivax *, coexists with *P. falciparum *in vast zones of the planet and it is prevalent in regions of Asia, Oceania, and Latin America where it is estimated to produce between 70 and 80-million clinical cases each year^2^. Mortality produced by malaria is higher in Africa, mainly in children younger than five years of age and in pregnant women infected by *P. falciparum *and although mortality is present to a lesser degree in infections by *P. vivax*, a significant number of lethal cases has been recently documented in high-transmission regions like India and Brazil^3^ ^,^ ^4^.

## Strategies and global programs of malaria control

Although the classical measures of malaria control, like early diagnosis, timely and efficient treatment, and mosquito control have contributed significantly to reducing the malaria distribution map^5^, it is currently considered that the development and possible application of malaria vaccines would contribute significantly and cost-effectively to reduce the impact of malaria in zones affected by the disease, and would favor its elimination in zones that currently have lower transmission. During the last decade, several global initiatives aimed at efficient malaria control have been developed including: the program denominated Roll Back Malaria (RBM), the Malaria Elimination Group (MEG), and, more recently, the Malaria Eradication Research Agenda (malERA). Additionally, the Global Fund (GF) has been, since 2002, perhaps the main funding source for malaria control Worldwide.

Until now, no malaria vaccine has been licensed for massive application in populations; however, rising evidence indicates the feasibility of developing vaccines. Firstly, in individuals exposed to malarial infection in endemic areas, the naturally acquired immunity accumulates progressively during the first two decades of life and results in decreased clinical severity of the disease and mortality^6^. Secondly, experimental immunization of non-immune volunteers with sporozoites previously attenuated through irradiation has demonstrated that up to 90% of the individuals vaccinated develop sterile protection against the experimental infection^7^. Thirdly, it has been shown that passive transfer of immunoglobulin from immune adults to naive volunteers eliminates the circulating parasites^8^. Additionally, it has been recently shown that it is possible to protect endemic communities from *P. falciparum, *at least partially, through immunization with an experimental vaccine, the RTS,S based on the* P. falciparum *CS protein^9^ *.*

## Importance of vaccines as control strategy

At least three levels have been contemplated of the *Plasmodium *cycle in which the parasite would be most susceptible to the immunological attack induced by a vaccine: the pre-erythrocytic stage (sporozoites and liver stages) and the asexual erythrocytic phase based on its capacity to stimulate humoral and cellular immune response. During the pre-erythrocytic stage, antibodies can inhibit invasion of sporozoites to the liver^10^ and, hence, prevent hepatic development of the parasite and the ulterior disease; cytokines like IFN-γ produced by T CD4+ and T CD8+ cells would contribute to halt intracellular development of hepatic schizonts^11^. During the erythrocytic stage, the presence of antibodies can, through different mechanisms, prevent invasion of the parasite to the erythrocytes^12^ and also, in red blood cells, oxygen radicals can destroy intracellular parasites^13^. A third level in which the parasite´s life cycle can be interrupted is the sporogonic phase, which occurs in the mosquito´s intestine. During this phase, it is possible to interrupt the fertilization process and ookinete invasion to the mosquito´s intestinal cells, preventing development of the parasite within the mosquito and, hence, its transmission to other susceptible individuals^14^.

Although vaccines must individually prove their efficacy, it is considered essential to focus efforts on generating formulations that include all the stages of the parasite´s cycle. Additionally, given the epidemiological distribution of malaria throughout the world, a functional vaccine against it must include components from at least the two most abundant species, *P. falciparum* and *P. vivax*. Accomplishing this aim in the near future is not easy because of the differential development of research on *P. falciparum* and on *P. vivax* . This review seeks to describe the use of high-performance tools and recent progress in identifying new antigen candidates for vaccines against *P. falciparum* and *P. vivax* during erythrocytic and pre-erythrocytic stages.

## Strategies to discover antigens with potential for vaccine development:

### Classical strategies

Vaccine production from inactive living, attenuated, or dead organisms, which have been employed to develop several of the vaccines for use in humans is not functional for diseases like malaria due to numerous factors like: contamination of the formulation with components from human cells, loss of immunogenicity, and difficulty in logistics for their production^15^ ^,^ ^16^. Thereby, the approach used during the last two to three decades to design a malaria vaccine has been based on identifying "subunits" of the parasite, such as complete antigens or their fragments, which have been mainly produced as synthetic peptides and recombinant proteins derived from sporozoite, merozoite, or gametocyte stages. Additionally, other methods have been tried like the production of vaccines from DNA and recombinant viruses. Numerous antigens, particularly from *P. falciparum* have been produced and analyzed in preclinical studies (on animals) in which their immunogenicity and lack of toxicity have been determined; essential conditions for their advance to the clinical development phase in humans. This process has given way to currently most advanced proteins being tested in clinical and preclinical phases (Table 1). Particular emphasis has been made on the *P. falciparum* circumsporozoite (CS) protein, which has reached maximum progress in its clinical development, recently accomplishing its analysis during Phase III clinical studies. This vaccine denominated Pf-RTS,S has demonstrated the capacity to induce protection against clinical and severe malaria in African children^9^, and although most recent studies registered protection of ~30%, the progress and learning accomplished during its analysis is of great value. The homologous protein in *P. vivax* has been analyzed in clinical trials in Phases I^17^ and, currently, a Phase II study is under way. Several other vaccines have reached clinical phases and are reviewed in available literature^18^ ^,^ ^19^.

**Table 1.**
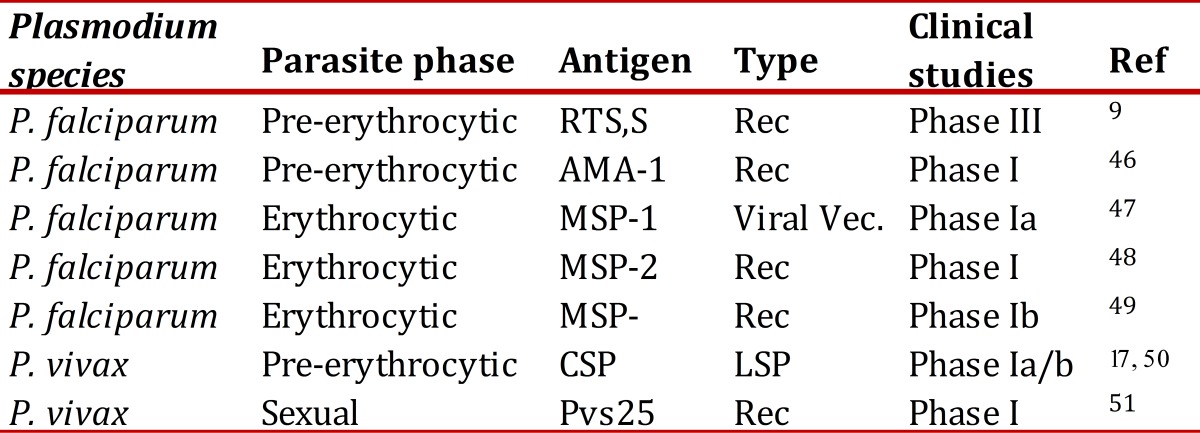
Description of malaria antigens in advanced phases of development, RTS,S: CS repetitive region, T-cell epitopes, hepatitis B surface antigen, AMA: apical membrane antigen, MSP: merozoite surface protein, CSP: Circumsporozoite protein, LSP: Long synthetic peptide, Rec: Recombinant, Vec: vector

## New strategies of antigen discovery

Use of the Expressed Sequence Tags (EST) technology permitted, during 2000-2003, describing the human genome and the genomes of multiple microorganisms of biological, agricultural, archaeological, and medical interest^20^ ^-^ ^22^. This vertiginous development permitted learning that parasites from the *Plasmodium* genus have genomes composed by between 5000 and 6000 genes. Availability of genomes from *P. falciparum, P. vivax* and from some other species, as well as progress in bioinformatics, genomics, and proteomics, are permitting the development of high-performance methods to select, clone/synthesize, and analyze a large number of proteins of these parasites, and it is probable that it accelerates development of malaria vaccines. Specifically, proteomic analyses have indicated that throughout the parasite´s life cycle at least 5,440 proteins are expressed by *P. falciparum* and 5,321 by *P. vivax* ^23^ ^,^ ^24^. Given this enormous multiplicity of proteins, it becomes more evident that the clinical immunity developed under natural and experimental conditions against human malaria is probably induced by multiple components of the parasite whose identification and analysis is only possible by using high-performance techniques.

## Proteomics-based tools for discovery of new vaccine candidates

As a result of this important progress, the development of high-performance technologies has emerged as great promise, and terms like transcriptomics, metabolomics, lipidomics, and proteomics are increasingly more common in biomedical literature, and it is expected that in the following years we will see the huge scope of technologies^25^. In light of the need to analyze the big volume of information produced on only one experiment based on high-performance technology, recently numerous tools have been developed for information analysis, which currently facilitate data mining and efficient development of complex studies employing "omic" technology.

## Microarrays of proteins

**Figure 1:**
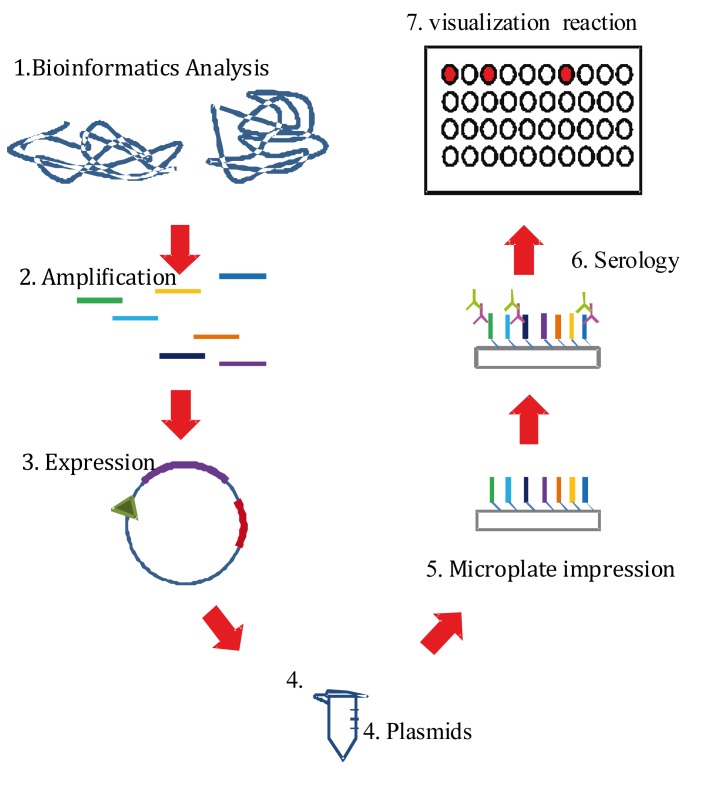
Production scheme of protein micro-arrays to identify antibody-target proteins

Consist of protein libraries assembled on a same format generated through cloning and expression of large fragments of the genome of microorganisms, in this case of *P. falciparum and P. vivax*, available to simultaneously conduct studies, for example, of reactivity with antibodies. In contrast to classical techniques that permitted analysis of individual genes and proteins, this proteomic technology permits simultaneous analysis of immunoreactive profile of hundreds of the organism´s significant proteins or of its fragments and represent one of the most attractive approaches for large-scale discovery of new vaccine candidates. One of the pioneer laboratories in malaria proteomics research is led by Dr. P. Felgner at the University of California (USA). His group developed a high-performance system for protein expression called "PCR Express", through which complete proteomes of any microorganism are generated^26^ (Fig 1).

Compared to conventional cloning methods in plasmid expression, transformation, and growth vectors in bacteria, "PCR express" offers various advantages for the production of transcriptionally active genes, given that these use up a large amount de time and require intensive labor, above all when seeking to simultaneously clone a large number of genes. The difference lies in that PCR express is based on a sound and practical approach for production transcriptionally active PCR fragments (TAPF) in two sequential PCR reactions. Technically, the process consists of two stages; a first stage uses gene-specific primers to amplify the gene of interest, while a second stage carries out nested PCR, which uses a mix of DNA fragments to add promoter and terminator sequences to each fragment. This makes the TAPFs equally active to super-coiled plasmid DNA, produced in in-vitro and in-vivo transfection assays and, hence, can be used as DNA vaccines^27^.

As in DNA vaccines and in recombinant viruses containing *Plasmodium* protein inserts previously described^28^, TAPFs can also be quickly transferred to plasmid vectors through homologous recombination, offering a high-performance cloning method that does not require using restriction enzymes or ligation reactions.

The TAPF technology has been used with different pathogens to evaluate serum antibody titers from people or animals, vaccinated or infected naturally, to identify antigens recognized by the immune system after vaccination or infection with such microorganism^29^.

Based on this technology, the most reactive antigens are selected for evaluation in different kind of studies, e.g., immunogenicity to determine their potential for vaccine development or to design immune-diagnostic methods. Until now, micro-arrays corresponding to ~30 microorganisms have been assessed, including viruses, bacteria, and pathogen parasites (Table 2).

**Table 2.**
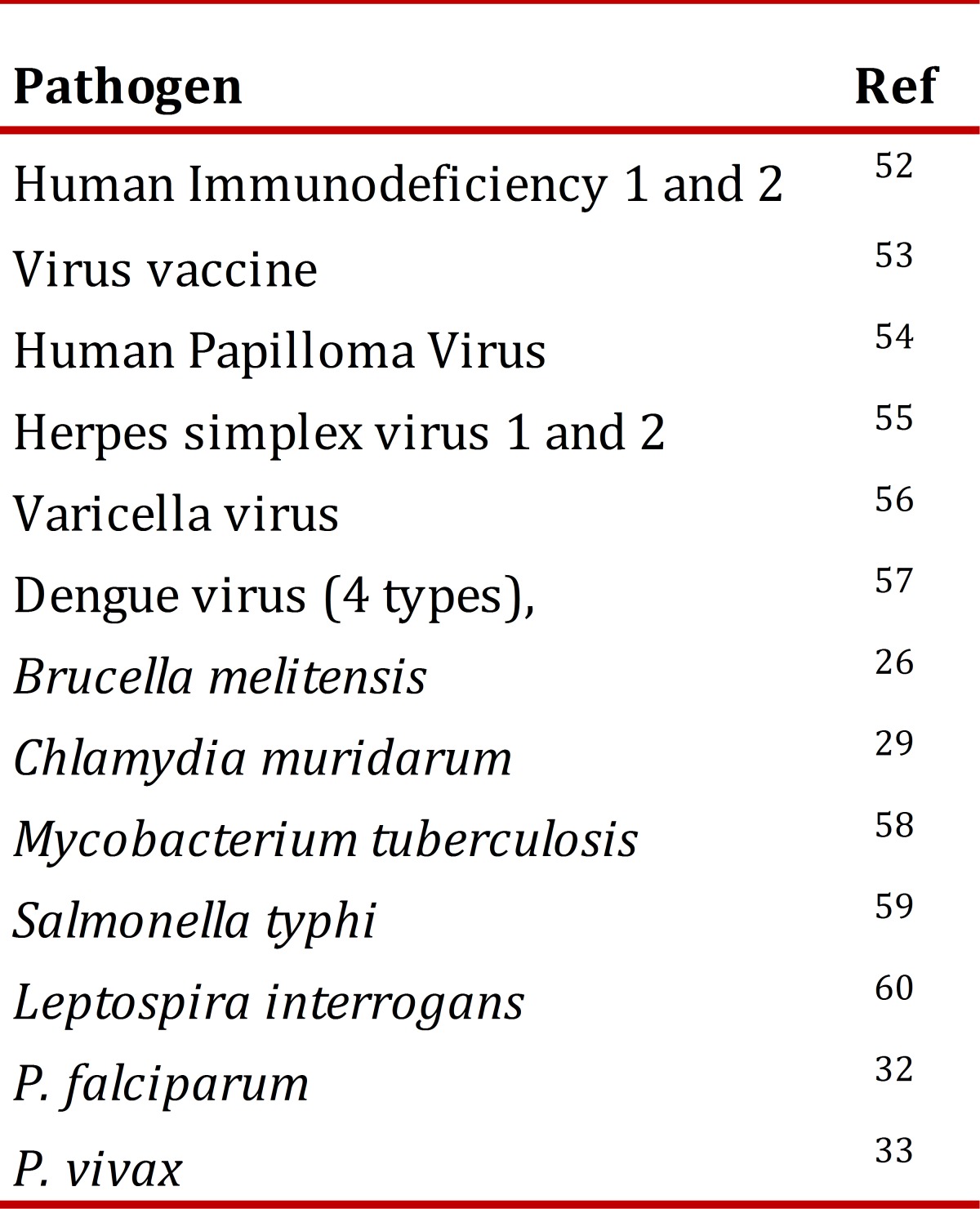
Microorganisms evaluated via micro-arrays

## Progress in the discovery of new Plasmodium antigens using protein microarrays

During the *Plasmodium* life cycle, over 5,000 proteins are expressed and it is not exactly clear which of these antigens mediate the clinical immunity and the protecive immunity observed on individuals from endemic areas or on individuals vaccinated with the parasite attenuated via irradiation^7^. Preliminary studies suggest that a large number of antigens are recognized^30^ ^,^ ^31^by the host´s immune system, highlighting the importance of identifying the repertoire of antigens and epitopes from the different development phases of the parasite implied in induction of clinical immunity. Recently, high-performance tools have been used to identify immunodominant antigens and preliminary definition of immunoreactivity profiles among groups of individuals with different levels of immunity to malaria^32^.

A first study selected a panel of 250 *P. falciparum* proteins chosen specifically to evaluate immune response in volunteers immunized with sporozoites attenuated via radiation, mainly in hepatic stages and with sporozoites, representing 4.75% of the totality of the genome, which were cloned, expressed and included in protein micro-arrays^32^.

The reactivity of these micro-arrays was then evaluated against serum samples from individuals vaccinated with irradiated sporozoites and compared to that of serum from individuals from endemic areas of Kenya, with different degrees of exposure to malaria. In this study, it was noted that subjects naturally exposed to malaria in Kenya reacted with greater intensity to a large number of antigens than individuals vaccinated with irradiated sporozoites. Also, among the group of subjects immunized with irradiated sporozoites, those protected against infectious challenge react with greater intensity and at a higher number of antigens than those unprotected. Additionally, it was noted that 56 of a total of 72 proteins that were the most highly reactive had not been previously characterized, which could represent a potential for development of malaria vaccines jo *P. falciparum* .

In another study held in Mali with serum samples from 220 individuals ranging in age between 2 and 10 years and between 18 and 25 years^31^, it was observed that the average number of proteins recognized by individuals exposed to the infection increased with age. Furthermore, it was found that reactivity in children increases dramatically during periods of high transmission of malaria. In contrast, the number of proteins recognized by the adults does not vary significantly during these periods. Finally, these analyses provided information about the patterns of reactivity against *P. falciparum* proteins based on the phases of the life cycle in which the proteins are expressed, as well as their sub-cellular location and other proteomic characteristics.

For *P. vivax* , few genes of the ~5500 encoded by the genome from the Salvador I (Sal I) strain^23^ have been assessed. Recently, 10 proteins from pre-erythrocytic stages were identified, which were widely recognized by serum from individuals from endemic regions of the Colombian Pacific Coast (n=60). 

These proteins were identified from a panel of 91 antigens evaluated in micro-arrays^33^ that are currently available for additional characterization.

Similar to the protein micro-arrays, the peptide micro-arrays are now being used for functional evaluation of proteins. Peptides are quite stable functionally, capable of maintaining their activity under most reaction conditions, which gives them advantages in application like micro-array. In general, depending on the peptide micro-array preparation method, these can be classified into: (a) in situ, or (b) synthesis followed by immobilization.

As the name implies, in situ micro-arrays are synthesized directly on the solid surface. The technique consists of dispensing a small volume of solutions containing amino acids and other coupling reagents to a designated point on a membrane.

In the second case, peptides are previously synthesized by using conventional equipment and methods and then nanoliters of peptide solutions are transferred to the solid surface. This approach is much more efficient because each peptide needs to be synthesized only once^34^.

Peptide micro-arrays have been used in identifying ligands or substrates of target molecules of interest, as well as in evaluating activity and enzyme and protein bonding^35^.

Peptide micro-arrays can be used, for example, to identify epitopes and evaluate the response of the immune system to different pathogens. Using this technology, Wiley et al., demonstrated that the recognition profile of different epitopes from a *P. vivax* protein changes en individuals vaccinated with the antigen formulated in different adjuvants^35^. 

## Using bioinformatic tools to select structural motifs in Plasmodium proteins

In spite of the great value of massively identifying immunodominant proteins, direct identification of relevant epitopes represents a high valuable complementary strategy. Many of the epitopes recognized by antibodies represent three-dimensional surfaces of an antigen molecule that precisely interact with the bonding surfaces of the corresponding antibodies; these epitopes can be linear or conformational. Linear epitopes are formed by a continuous sequence of amino acids, while conformational epitopes depend on the position of the amino acids in the protein´s three-dimensional structure, which is determined by the combination of its alpha (α) helix, folded beta (β) sheet, or β coil structures. It has been shown that α double-helix structures contain abundant epitopes from B cells that have proven to be targets of Plasmodium growth inhibiting antibodies^36^ and other pathogen agents^37^. Additionally, it is estimated that most B epitopes are structural and that only 10% of the antibodies induced by immune response are aimed against linear epitopes^38^.

**Figure 2:**
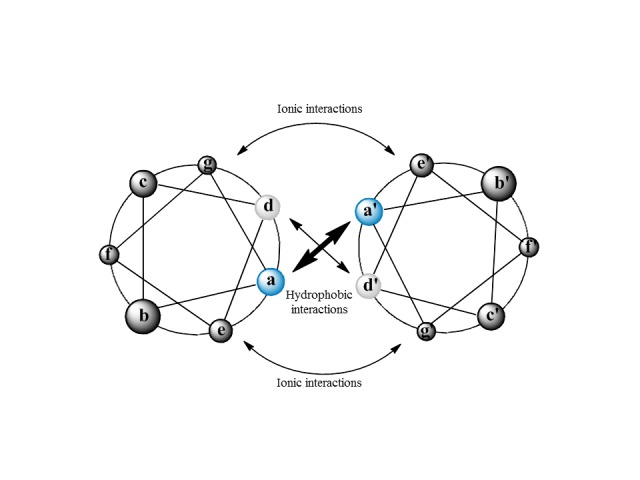
Schematic representation of alpha double-helix motifs

Production of protein fragments containing conformational epitopes is of great importance, but it represents a huge synthesis technical challenge^39^. Fragments of synthesized proteins need to acquire stable structures that mimic the native structure and, hence, can be recognized by antibodies. Further, the challenge exists of identifying such fragments in protein sequences throughout the genome/proteome. Recently, specific bioinformatic algorithms have been developed to select sequences containing α double-helix motifs. These motifs form stable structures characterized by the presence of repetitions from seven residues from amino acids (abcdefg) with hydrophobic residues located in positions a and d, and hydrophilic residues in the remaining positions (Fig 2), which are generally monomorphic and react with antibodies that are reactive with the native form of the native protein. These structures are easily identifiable with bioinformatic tools^40^, which significantly reduces antigen selection time.

Recent progress in chemical synthesis techniques have permitted the production of 95 fragments of 30-40 amino acids of length corresponding to *P. falciparum* proteins, which contained α double-helix motifs. All the antigens selected were evaluated to determine their antigenicity by using a panel of serum from donors from endemic zones^41^, which permitted identifying ~70 new proteins with variable length between 200 and 10,000 amino acids. Thereafter, the immunogenicity of some of these fragments was evaluated in murine models. Functional assays conducted using specific antibodies against the different fragments, demonstrated their capacity to inhibit in vitro development of the parasite in antibody-dependent inhibition trials (ADIT), and it was also demonstrated that these structures are highly conserved^42^. A Phase 1 clinical trial is currently analyzing fragment P27A of 104 amino acids derived from the *P. falciparum* protein PFF0165c selected by using this methodology^43^; in addition, the immunogenicity of the Pf-P181 polypeptide, also selected through this methodology and which contains fragments from three different proteins bound by a nonimmunogenic connector (diethylene glycol), was recently evaluated during preclinical trials^44^ ^.^

The same technology is being currently used in identifying *P. vivax* antigens. A total of 52 peptides containing α double-helix motifs were recently selected from 150 proteins of *P. vivax* erythrocytic stages. Fragments of variable lengths between 30 and 50 residues were synthesized by using F-moc solid phase chemistry and used to determine their antigenicity by comparing their reactivity with serum from individuals naturally exposed to malaria in hyper-endemic areas of Papua New Guinea (PNG) (District of Maprik) and from areas of medium and low transmission in Colombia (Tumaco and Tierralta). In general, higher reactivity was observed when using serum from individuals from PNG; a total of 10 fragments have been preselected because of their high reactivity with serum from PNG and from Colombia and they are being used in immunogenicity assays in mice.

## Conclusions

The large number of proteins produced by *Plasmodium* and their great diversity demand the use of high-performance tools for their identification, production, and analysis. During the last decade, important progress in bioinformatics and biotechnology has permitted the construction of micro-arrays of proteins produced via recombinant technology or as synthetic peptides, which have permitted identifying over 400 new antigens from pre-erythrocytic and erythrocytic phases with possible functions in the natural immunity acquired for *P. falciparum and P. vivax* . However, although this progress permits studying more efficiently the response profile of antibodies associated to immunity acquired naturally or through vaccination (antigenicity), immunogenicity analyses of proteins or epitopes selected still require in-vivo models which are not currently scalable. The important progress in the study of immune response against Plasmodium through high-performance methods now generates exceptional conditions for identifying new vaccine candidates for their clinical development.
